# Providing Psychological Therapy Support and Improving Postdischarge Data Collection: Preliminary Evaluation of the “Paddle” App

**DOI:** 10.2196/68671

**Published:** 2026-02-24

**Authors:** Graham R Thew, Michelle Lee, Ineke Wolsey, Fiona Giles, Emma Hayden, Grace Jell, Jamie Peacock, John Pimm, Joanne Ryder, Alison Salvadori, David M Clark

**Affiliations:** 1 Department of Experimental Psychology University of Oxford Oxford United Kingdom; 2 NHS Buckinghamshire Talking Therapies Oxford Health NHS Foundation Trust High Wycombe United Kingdom; 3 NHS Oxfordshire Talking Therapies Oxford Health NHS Foundation Trust Oxford United Kingdom; 4 Charlie Waller Institute School of Psychology and CLS University of Reading Reading United Kingdom; 5 Brighter Futures Together CIC Reading United Kingdom; 6 NHS Berkshire Talking Therapies Berkshire Healthcare NHS Foundation Trust Reading United Kingdom

**Keywords:** anxiety, app, cognitive behavioral therapy, depression, NHS Talking Therapies, outcome measurement

## Abstract

**Background:**

Clinical trials of psychological therapies such as cognitive behavioral therapy typically show sustained posttreatment effects. However, less is known about individuals’ outcomes following treatment in routine practice, and additionally, patient representatives have highlighted a need for better postdischarge support.

**Objective:**

The “Paddle” app aims to address these issues. Paddle allows patients to store their therapy materials, reflect on sessions, and monitor their well-being during and after treatment, completing outcome questionnaires and monthly reviews.

**Methods:**

Study 1 evaluated patients’ use of Paddle during treatment in routine practice. Patient and therapist surveys explored feasibility, acceptability, and helpfulness. Study 2 examined the feasibility of using the app post discharge, inviting users to submit monthly questionnaires for 6 months, and seeking feedback via survey.

**Results:**

Our findings indicate that Paddle was feasible to implement with few technical problems. Although not all patients wanted to use an app in Study 1, uptake was 66% (111/168) and users found it acceptable and helpful for organizing and remembering therapy information. Most reported using the app on a weekly or fortnightly basis. In Study 2, a total of 321 patients downloaded and used the app at least once, of whom 49% (156/321) submitted follow-up data. Of those who reliably improved during treatment, 73% (86/118) remained so throughout the 6-month follow-up period. Among all users, 20% (31/156) showed further reliable improvement at least once compared to their end-of-treatment score. We introduce the concept of “reliable relapse,” which occurred for 36% (32/89) of users who had reliably recovered during treatment, highlighting that some experience fluctuations or deterioration. Feedback highlighted Paddle’s value in helping people self-monitor and prioritize their well-being after treatment, with 81% (50/62) suggesting a postdischarge follow-up period longer than 6 months would be helpful.

**Conclusions:**

These preliminary findings suggest that Paddle shows promise in supporting patients to collate therapy resources and monitor their well-being during and after treatment. It may help to improve rates of follow-up data collection, which warrants further investigation.

## Introduction

Psychological therapies, such as cognitive behavioral therapy (CBT), have a strong evidence base demonstrating their efficacy in the treatment of anxiety and depression [1-3]. They are recommended as first-line treatment options by health guidance organizations such as the National Institute for Health and Social Care Excellence [4,5], due to there being stronger evidence for sustained gains compared to other treatments such as medication [6]. Evidence from randomized controlled trials suggests that, on average, the clinical gains made during treatment are well-maintained after treatment ends, with studies typically showing sustained effects in follow-up assessments 12-24 months posttreatment [7-9]. However, although improvements are maintained on average, some people show further gains, and some people show deterioration during the follow-up period.

Services providing such therapies within routine clinical practice are often limited in their ability to provide posttreatment support to patients, and to monitor the outcomes of those after they have been discharged. In England, data from the National Health Service Talking Therapies programme for anxiety and depression (NHS TT) in 2022-2023 showed that only 1.3% of patients received a follow-up appointment after they had been discharged [10]. This is perhaps not surprising because these services are not commissioned to collect follow-up data, but it does highlight that we know little about how such patients progress after leaving treatment, and that efforts to increase postdischarge data collection may be valuable for our understanding of the maintenance of treatment gains in routine practice.

A small number of studies have examined posttreatment relapse rates in cohorts of patients who had recovered following low-intensity CBT. Observed relapse rates included 29% in the first 9 months [11] and 53% within 12 months [12]. Factors increasing the likelihood of relapse have included residual depression symptoms at the end of treatment, younger age, unemployment, and nonlinear treatment responses [12,13]. Such studies provide helpful indications about which patients may be at greater risk of relapse. They also highlight areas where further research is needed. For example, studies to date have focused only on low-intensity treatment, so our understanding of posttreatment outcomes across whole services (which include high-intensity treatments and sequential low- and high-intensity interventions) is limited. Studies to date have focused only on patients who recovered during treatment, meaning less is known about those who showed reliable improvement in symptoms but did not fully recover. Finally, the time-to-relapse analyses used in most studies to date place less focus on what happens after a relapse, suggesting there is more to understand about the duration of relapses and the frequency of fluctuating symptom trajectories in this period. Furthering our knowledge of patient outcomes in the posttreatment period is an important step toward better tailoring of treatments or developing targeted posttreatment support to those who may particularly benefit. This might reduce the need for patients to return to services [14], enhancing clinical effectiveness and the efficient use of services’ resources.

Importantly, the need to improve posttreatment support and prevent relapse has also been identified as a priority by patients. Patient and public involvement (PPI) on this topic in the Thames Valley region led to the development of the “Staying Well” guidance and support package that has been recommended for use as a component of low-intensity interventions in NHS TT services (materials available at Oxford Centre for Anxiety Disorders and Trauma, 2024) [15]. PPI contributors also highlighted that therapies such as CBT often result in a large number of materials, such as worksheets, diaries, flashcards, pictures, and diagrams, and that it can be challenging to organize and store these in ways that are accessible and useful both during therapy and afterward. Many had kept a folder or notebook but found that if they were out or away from home, they were not able to remind themselves of the key points or reminders they had generated from treatment. As one contributor put it, “a folder is no good if you are having a panic attack on the bus.”

These discussions led to the development of an app (“Paddle”; Global Initiative Ltd) as a collaboration between PPI contributors, regional services, clinicians, and software developers [16]. Digital tools have been suggested as a helpful method to enhance the delivery of psychological therapies [17] and their role in preventing relapse has been identified as a priority research area [18]. Paddle is a secure, personal storage app in which a patient can add and organize their materials from therapy, as well as keep notes or reflections about their sessions. The app also includes easily accessible emergency and crisis information. Finally, Paddle allows patients to complete routine outcome measures within the app, sending these results directly to services, and presenting graphs and information about these questionnaires to the patient, to support them in monitoring their own well-being both during and after treatment. It is hoped that Paddle represents a useful and effective adjunct to standard psychological therapy interventions, which may support patients in organizing, accessing, and remembering their therapy materials during and beyond treatment.

This paper reports the findings of 2 studies evaluating the use of Paddle as part of routine service provision in 3 NHS TT services. The research questions were as follows:

1. Is Paddle feasible to use alongside standard psychological therapy interventions?
2. Do patients and therapists find the app acceptable?
3. Is it feasible to use Paddle to collect outcome data following the end of treatment?
4. What are patients’ views on using Paddle to monitor their own well-being after discharge?

## Study 1

### Methods

#### Design

This was a survey-based study of participants starting treatment in the participating NHS TT services (Berkshire, Buckinghamshire, and Oxfordshire) whose therapist introduced and discussed Paddle with them. Both low- and high-intensity interventions were included.

#### App Description

Paddle is a prescribed (invite-only) app available to patients receiving treatment for anxiety and/or depression in NHS TT services. It is designed to be used solely by the patient as an adjunct to their psychological therapy intervention at low or high intensity, allowing them to add, organize, and store their therapy-related information in one secure location. These materials are then easily accessible for the patient both during treatment and afterward. It does not therefore deliver therapy content but is an adjunctive tool designed to support psychological interventions such as CBT. Paddle meets all relevant technical and data security standards (DCB0129 of the NHS Data Coordination Board, ISO [International Organization for Standardization] 9001, and ISO 27001). Since the completion of the studies described here, Paddle has been approved and registered as a Class I medical device.

**Figure 1:**
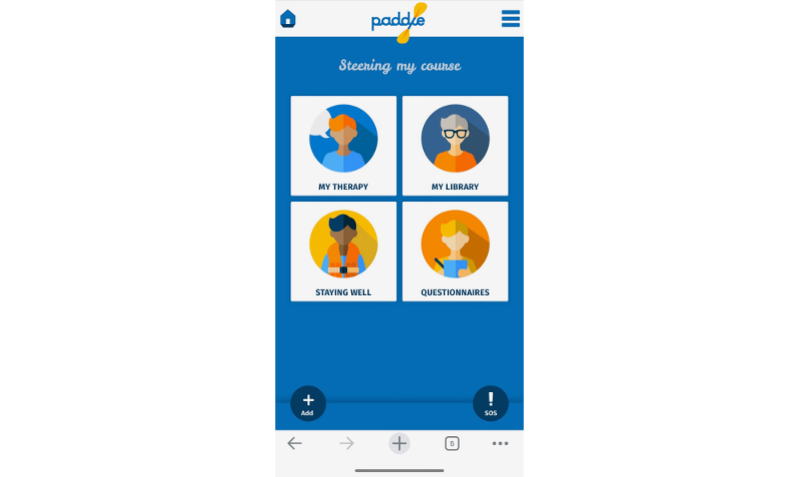
Screenshot of a phone showing the Paddle app home page.

During Study 1, the app allowed patients to upload information to 3 areas: “My Therapy,” where patients could enter notes, actions, reflections, and materials from their sessions; “My Library,” where patients could develop a bespoke set of resources, including images, audio files, documents, websites, and telephone numbers; and “Staying Well,” where patients could add information and resources for emergencies, setbacks, or difficult moments (eg, a safety plan, relapse prevention plan, or other key points to remind themselves of during and after treatment). In addition, an “SOS” section of the app allows patients to easily access their most important resources when needed. Figure 1 shows a screenshot of the app homepage [16].

#### Participants

A group of 18 therapists (12 psychological well-being practitioners and 6 CBT therapists) across the 3 participating services attended a half-day in-person training session to learn about the aims, features, and functionality of the app, how to introduce Paddle to their patients, and how to generate an app invite for patients.

These therapists then discussed Paddle with eligible patients on their caseload. The only exclusion criteria were patients using an internet-based platform to undertake treatment, for example, SilverCloud (SilverCloud Health) [19], iCT-SAD (internet-delivered cognitive therapy for social anxiety disorder) [20] or iCT-PTSD (internet-delivered cognitive therapy for posttraumatic stress disorder) [21], or those attending a brief psychoeducation-only group.

Paddle was discussed with 203 patients, and 168 (83%) agreed to be sent an invite to download the app. Of these, 111 (66%) downloaded and logged into the app at least once.

#### Procedure

Therapists from the participating NHS TT services who attended the training were asked to introduce the Paddle app to each new patient during the first or second treatment session between October 2019 and February 2020. Patients were signposted to the Paddle website for further information and sent a sheet of frequently asked questions about the app. Therapists then asked patients if they wished to be sent an invite to download and use the app. Those who agreed and downloaded the app were then able to use it as they wished for the duration of their treatment. Therapists were not required to prompt discussion or use of Paddle but could do so if clinically relevant.

At the point of discharge from treatment, the therapist invited the patient to complete an online survey (using the Qualtrics survey platform; Qualtrics LLC). All 203 patients were invited. A branching survey design was used to present questions relevant to respondents. For those who had requested an invite to download Paddle, questions were asked about their use of and experience with the app. For those who declined to be sent an invitation, they were invited to share feedback about their reasons for doing so. Response types on the survey therefore included Likert and frequency response scales, multiple choice, and free-text questions.

At the end of the study period, the participating therapists were also invited to complete an online survey to gather their impressions of Paddle based on their interactions with patients, and record any technical issues they had experienced when sending invites.

#### Data Analysis

Responses to both the patient survey and therapist survey were analyzed descriptively.

#### Ethical Considerations

The study was registered and approved as a pilot service evaluation project with the research and development departments of the participating NHS trusts (Oxford Health NHS Foundation Trust and Berkshire Healthcare NHS Foundation Trust). Ethics approval was sought and granted for this process within the University of Reading (Reference UREC 19/02). All participants provided consent to participate and for their data to be used to support service evaluation and improvement. Any identifiable data was removed prior to analysis. Compensation was not provided.

### Results

#### Patient Survey

A total of 68 responses were received, giving an overall response rate of 33% (68/203). Respondents comprised 51 people who had logged in at least once and 17 who declined to be invited to Paddle. The response rate of those who had logged in was therefore 46% (51/111), and of those who declined was 49% (17/45). There were no responses from patients who had received an invite to download the app but had not logged in.

The age range of Paddle users, as self-reported on the survey, was 16-59 years, with the most common reported age range being 20-29 years. The age profile of nonusers was older, ranging from 20 to 69 years, with the most common reported age range being 60-69 years. Overall, 70% of this sample were 50 years or older, compared with 10% of the sample who had chosen to download the app.

Paddle users all reported using other apps in everyday life, with 69% (35/51) using other health-related apps. On average, they rated their confidence in using health-related apps as 78/100 (SD 11.61). In contrast, only 12% (2/17) of the nonusers group reported using other apps, with only one person using a health-related app. Mean confidence was 10/100 (SD 2.89).

The majority (45/51, 88%) of Paddle users indicated that they had downloaded the app because their clinician had recommended it, and 63% (32/51) because they wanted to have their therapy materials together and to hand. Paddle nonusers gave 2 main reasons for declining to use the app: a lack of confidence with this type of technology and concerns about information security.

Paddle users reported using all sections of the app, including “My Therapy” (46/51, 90%), “My Library” (36/51, 71%), and “Staying Well” (40/51, 78%). Most reported using the app on a weekly (29/51, 57%) or fortnightly (12/51, 24%) basis during treatment, which likely reflected use in relation to each therapy session. Some users used the app more than once per week (8/51, 16%) and some less than fortnightly (2/51, 4%). Users’ agreement ratings with statements about Paddle are shown in Table 1. These reflected positive feedback regarding the feasibility, acceptability, and helpfulness of the app. Free-text comments indicated that users liked that this app had been developed by patients for patients, and that everything was in one place to refer back to. Various comments indicated the app had also been helpful in organizing users’ thinking: for example, “Summarising the sessions in the app helped me to focus and remember the techniques,” “It was really helpful writing a summary from each session to get my thoughts together,” “It’s helped me to organise my thoughts, I can look back on this when I need to,” “It helped me to think about what I needed to focus on and what I’d learnt.” Comments also indicated that users felt the app will be a helpful resource to use following the end of treatment.

**Table 1:** Number of Paddle users agreeing with statements about the app.

Statement	Strongly disagree	Disagree	Neutral	Agree	Strongly agree
Paddle has helped me make the most of treatment	0	1	13	20	17
Paddle has been useful to me	0	1	12	19	19
Paddle has been straightforward to use	0	0	13	22	16
I’ve liked using Paddle	0	1	14	19	18
I plan on using Paddle now treatment has ended	0	5	12	23	11
I would recommend Paddle to others having treatment for anxiety or depression	0	1	14	21	15

#### Therapist Survey

Eleven responses to the therapist survey were received, a response rate of 61%. Responses came from 7 psychological well-being practitioners and 4 CBT therapists. Therapists’ comments highlighted patients’ interest in the app and willingness to receive an invite to download it. They noted the value of discussing it together with the patient (“Explaining about Paddle in the session is helpful – especially if there are questions”)*.* Consistent with findings from the patient survey, age and technological confidence were reported as factors that might be associated with not wanting to use the app (“They might not be used to using apps”; “It’s been my older clients who are more hesitant”)*.* No major technical problems or barriers to use were reported. Therapists’ impressions of their patients’ experiences using the app alongside treatment were positive (“I had one client who said how useful it was to have a place to store everything together,” “Clients seem to like it and are finding it useful,” “I’ve had very positive feedback about it,” “Clients were definitely using it between sessions to record info and help them prepare for subsequent sessions”)*.*

### Discussion

#### Principal Findings

The findings of this study provided an initial indication that it was feasible for therapists to introduce and discuss Paddle with their patients, and for patients to download and use the app during treatment. No major technical issues were observed. Acceptability was indicated by the high rate (168/203, 83%) of those willing to receive an invite to the app, and of those who downloaded and used the app at least once (66% of those invited, 111/168). Most patients reported using the app regularly during treatment. The positive feedback provided an initial indication that the app was valued and perceived as helpful by the majority of respondents, though we cannot say whether nonrespondents had similar views. The findings also suggest that not everyone found the idea of using an app alongside therapy to be helpful, which may be associated with technological confidence and concerns about information security.

These initial findings that the app could be implemented during treatment and that it could be a helpful tool for patients indicated that progression to the next phase of development was warranted. This involved adding the functionality to complete outcome questionnaires within the app.

## Study 2

### Methods

#### Design

This was a single-cohort observational study, examining (1) whether the app could be used to collect outcome data following discharge and (2) whether users found using the app helpful to self-monitor their well-being in this phase. Unlike in Study 1, we invited patients at the point of discharge to download and use Paddle to support their posttreatment well-being. All features of the app were available, including the functionality to complete routine outcome measures within the app. Patients were invited to complete these questionnaires on a monthly basis for the first 6 months following discharge.

#### App Description

The app was identical to that used in Study 1, with the addition of a “Questionnaires” area, which allowed users to complete the same routine outcome measures they had used during treatment. It also provided users with a brief explanation of each questionnaire and what it measures. After completing a set of questionnaires, users could view graphs and tables of their scores over time, allowing them to compare these with their scores at the start and end of treatment. Based on the trajectory of the 3 most recent scores on each questionnaire, and the current score in relation to the standard clinical caseness threshold for that measure (described in National Collaborating Centre for Mental Health, 2018) [22], Paddle provided brief feedback and using short question prompts, encouraged users to reflect on what had been going well or less well and to continue building on strategies they were finding helpful, referring to their “Staying Well” materials as required.

Users’ submitted questionnaire data were automatically integrated with the electronic care record used by the relevant NHS TT service. It was explained to users that because they were not currently under the care of the service (having been discharged), the scores they submit to the service were for information only, and if they wanted to access additional support, they should contact the service or other health care providers as normal.

One of the routine outcome measures, the Patient Health Questionnaire 9-item version (PHQ-9) [23], contains an item relating to risk of suicide or self-harm. It was agreed with services that in the course of looking to improve follow-up data collection after discharge, there is a responsibility to respond appropriately if significant risk is indicated. Based on discussion with clinicians, service managers, and patient representatives, we established the following procedure for this study. If any risk was indicated on the PHQ-9 (ie, a response >0 on the self-harm and suicidality item), the app presented 4 brief additional questions to the user, exploring plans, preparations, behavioral intent, and protective factors. All PHQ-9 data, plus any responses to the additional questions, were sent directly to the relevant service. A nominated clinician in each service reviewed these on a regular basis in conjunction with the clinical notes. Based on their clinical judgment and local service procedures, the clinician would contact the patient if necessary to conduct further risk assessment and management. Users were informed that services are not able to respond to mental health crises and provided with a list of emergency and crisis service details for them to contact if needed.

#### Participants

Patients were eligible if they reported a main problem of depression or an anxiety disorder and had received at least 2 therapy sessions, at low- or high-intensity. NHS TT services deliver National Institute for Health and Care Excellence (NICE)-recommended therapies, most commonly CBT but also approaches such as counselling or interpersonal therapy. They were not eligible if their last course of treatment prior to discharge used an internet-based treatment platform, because these treatments typically allow users to continue to access the treatment material for a fixed period after discharge. Patients were also not eligible if they were being referred on for further treatment in another service.

Across the 3 participating NHS TT services, 7714 patients were discharged within the 13-week study recruitment period (October 2021 to January 2022), of whom 4063 were eligible and invited to participate. Of these, 321 patients (8%) downloaded and used the app at least once.

#### Procedure

All clinical staff received a brief training webinar explaining about Paddle and how to introduce it to eligible patients. Where possible, the treating therapist introduced and explained Paddle during the final treatment session and sent the invitation to download the app. However, in most cases, this was not possible, so a designated clinician in each service reviewed the list of recently discharged patients and sent the app invite by email.

Paddle users were free to use all areas of the app as they wished. They received a monthly email invitation to submit their questionnaires, with a reminder 1 week later. At the end of their 6-month period post discharge, anyone who had logged into Paddle at least once was invited to complete a feedback survey via the Qualtrics survey platform. Questions focused on the use of the questionnaire area of the app, and the experience of using Paddle post discharge.

#### Data Analysis

App usage, clinical outcomes, and user feedback were analyzed descriptively. The demographic and clinical representativeness of app users was analyzed using 2-tailed *t* tests and chi-squared tests.

#### Measures

As is standard in NHS TT services, all participants were given standardized and well-validated measures of depression (PHQ-9; Kroenke et al [23]), generalized anxiety (Generalized Anxiety Disorder 7-item version; GAD-7; Spitzer et al [24]), and general functioning (Work and Social Adjustment Scale; WSAS; Mundt et al [25]). Where a participant’s treatment focused on a specific anxiety disorder, Paddle administered a relevant additional measure, matching that which was used during treatment. Anxiety disorder specific measures (ADSM) used in this study were Health Anxiety Inventory [26], Obsessive Compulsive Inventory [27], Posttraumatic Stress Disorder Checklist for *DSM-5* (*Diagnostic and Statistical Manual of Mental Disorders* [Fifth Edition]) [28], Panic Disorder Stress Scale [29], Patient Health Questionnaire 15 item [30], and Social Phobia Inventory [31]. These measures are also well validated and used as standard within NHS TT services [22]. Participants were also asked to provide their employment status and indicate their use of health care services in the past month.

#### Classification of Clinical Outcomes

Clinical outcomes were categorized following standard NHS TT definitions, which are calculated based on changes in the PHQ-9 and GAD-7 scores. Where ADSM scores are available, these are used in place of the GAD-7. Reliable improvement is indicated where scores on the PHQ-9 and/or GAD-7/ADSM decrease by an amount greater than the reliable change index for the measure, and neither measure shows a reliable increase. Reliable deterioration is indicated by an equivalent increase in scores, with neither measure showing a reliable decrease. Reliable recovery is indicated where a patient starts treatment above the clinical caseness threshold on the PHQ-9 and/or the GAD-7/ADSM, shows reliable improvement, and has an end score below caseness on both measures. For further details, see the NHS TT manual [22].

For this study, we developed the concept of “reliable relapse,” which is the conceptual opposite of reliable recovery. Reliable relapse is indicated where a patient is below caseness on both PHQ-9 and GAD-7/ADSM, shows reliable deterioration, and has an end score that is above caseness on either or both measures. By requiring that scores must have shown a deterioration that is clinically meaningful and more than might be expected by measurement error alone (ie, reliable deterioration), this definition avoids classifying a small change from just below to just above the clinical cutoff as a relapse.

#### Ethical Considerations

The study registration and ethical considerations were identical to Study 1. The study was registered and approved as a pilot service evaluation project with the research and development departments of the participating NHS trusts (Oxford Health NHS Foundation Trust and Berkshire Healthcare NHS Foundation Trust). Ethics approval was sought and granted for this process within the University of Reading (Reference UREC 19/02). All participants provided consent to participate and for their data to be used to support service evaluation and improvement. Any identifiable data were removed prior to analysis. Compensation was not provided.

### Results

#### Use of the App to Complete Questionnaires

Of the 321 patients who downloaded and used the app at least once, 156 (49%) submitted at least one set of questionnaires and thus provided follow-up data. No major technical problems occurred. Patterns of data completion across the 6-month follow-up period were variable: 30 (19%) users provided data in all 6 months, 17 (11%) provided data on 5 occasions, 19 (12%) on 4 occasions, 17 (11%) on 3 occasions, 20 (13%) on 2 occasions, and 53 (34%) on 1 occasion. ADSMs were used by 17% of those who submitted questionnaires.

A total of 487 PHQ-9 questionnaires were completed during the study, of which 78 (16%) included a score of 1 or more on the risk item, and 23 (5%) showed an increase in score compared to the last available datapoint. Upon review of the PHQ-9 and additional risk questions by the designated clinician in each service, seven users were contacted for further risk assessment and management as appropriate.

#### Representativeness of App Users

We compared the demographics of Paddle users with those who were invited but did not log in. No significant differences were observed with respect to age (*t*_4060_=–0.031; *P*=.98, or gender: χ^2^_2_=1.5; *P*=.47). The proportion of those from non-White ethnic backgrounds (ie, selecting options other than “White British,” “White Irish,” and “White Other”) was lower among Paddle users (10.26%) compared to nonusers (14.36%; χ^2^_1_=3.9; *P*=.049). The same pattern of results was shown when comparing Paddle users with all other people who were discharged within the study recruitment period: age (*t*_7711_=–0.084; *P*=.93; gender: χ^2^_2_=1.8; *P*=.41; ethnicity: χ^2^_1_=6.5; *P*=.01).

To compare treatment characteristics between Paddle users who submitted follow-up data and those who did not, we compared the number of treatment sessions received. Users who provided follow-up data had received a greater number of sessions (mean 15.05, SD 10.89) compared to those who did not (mean 11.64, SD 7.85; Mann-Whitney *U*=9858.50; *P*=.004).

#### Clinical Outcomes

Clinical outcomes were explored for the 156 Paddle users who submitted follow-up questionnaires. We started by examining their clinical outcomes during treatment. The rate of reliable improvement during treatment was 76% (118/156), and reliable deterioration was 4% (7/156). Reliable recovery was shown by 61% of users during treatment (89/146 users who started treatment above caseness). The mean PHQ-9 score was 14.45 (SD 5.66) at the start of treatment, and 7.24 (SD 5.18) at the end of treatment. The mean GAD-7 score was 12.47 (SD 4.97) at the start of treatment, and 6.21 (SD 4.57) at the end of treatment.

An average of the PHQ-9 and GAD-7 scores provided by each user during the 6-month follow-up period was calculated as an indicator of symptom severity across this phase. The mean PHQ-9 score during follow-up was 8.40 (SD 5.21), and the mean GAD-7 score was 7.31 (SD 4.99). Paired *t* tests indicated that the follow-up scores were an average of 1.2 points higher on the PHQ-9 compared to the end of treatment (*t*_155_=–3.81; *P*<.001, Cohen *d*=0.31) and 1.1 points higher on the GAD-7 (*t*_154_=–3.85; *P*<.001; Cohen *d*=0.31). Although significant, the observed effect sizes suggested these increases were considerably smaller than the decreases seen when comparing the follow-up scores to the start of treatment scores (PHQ-9 *t*_155_=13.62; *P*<.001; Cohen *d*=1.09; GAD-7 *t*_154_=12.01; *P*<.001; Cohen *d*=0.96).

We then looked at individual change trajectories over the follow-up period, comparing each monthly follow-up score to the start of treatment score. First, we examined the 118 users who had reliably improved during treatment. This showed that 86 (73%) users stayed reliably improved across the 6-month period, 26 (22%) had at least one score that no longer reflected reliable improvement, and 4 (3%) showed reliable deterioration at at least one point. Two users (2%) had insufficient data for this analysis. The results also showed that of the 29 people in this group who had reliably improved but not reliably recovered during treatment, 5 (17%) went on to demonstrate reliable recovery during the follow-up.

Second, we examined those who had not reliably improved during treatment (n=38). We found that 7 users (18%) went on to reliably improve or reliably recover and remained so throughout follow-up. Three (8%) users showed reliable improvement or reliable recovery that was not sustained. Nine (24%) users showed no reliable changes in score throughout follow-up, and the remaining 19 users (50%) showed reliable deterioration at any point.

Third, we examined the group of users who had reliably recovered during treatment (n=89). There were 46 (52%) users who remained reliably recovered throughout the 6-month period, and a further 20 (22%) who remained either reliably recovered or reliably improved at each follow-up timepoint. Conversely, 18 (20%) users had at least one score that no longer reflected reliable improvement, and 4 (4%) showed reliable deterioration at at least one follow-up occasion. One (1%) user had insufficient data for this analysis. For this group, we also examined rates of reliable relapse by comparing each monthly follow-up score to the end-of-treatment score. This showed that 32 users (36%) showed a reliable relapse at any point during follow-up. In 9 of these instances, subsequent available data showed that the relapse was not sustained.

Finally, we examined changes within the follow-up period by comparing each monthly follow-up score to the end-of-treatment score for all cases (n=156). This showed that compared to when users ended treatment, 11 (7%) showed reliable improvement at every available timepoint during follow-up. Nine users (6%) showed a series of follow-up scores that reflected either reliable improvement or stability (ie, no reliable change compared to the end of treatment). Moreover, 72 (46%) showed stability at each available timepoint. In addition, 29 (19%) showed a series of follow-up scores that reflected either stability or reliable deterioration, and 23 (15%) showed reliable deterioration at every available timepoint during follow-up. There were 11 (7%) users with a fluctuating series of scores that included both reliable improvement and reliable deterioration, and 1 (1%) user with insufficient data for this analysis.

#### Employment and Health Care Use

There were 109/156 (70%) Paddle users who were in employment at the start of their treatment. Of these, 102 (94%) remained employed across the treatment and follow-up period (1 moved to sickness or disability benefits, 2 experienced a temporary period of unemployment, 2 became students, 1 retired, and 1 was unemployed at the end of follow-up). There were 11/156 (7%) users who were unemployed at the start of their treatment, 7 (64%) of whom moved into employment by the end of treatment, and remained in employment or study throughout the follow-up period.

Of the 153 users who provided information on their health care use at any point during follow-up, 45 (29%) reported accessing further health care services in the month prior to completing the questionnaire. Examples included general practitioner support, voluntary helplines, private psychological therapy, or other postdischarge support from NHS TT, such as online peer support (Browne et al [32]) or an additional booster session.

#### Patient Survey

Of the 321 people who had used Paddle at least once, 62 (19%) responded to the feedback survey. Most reported they had downloaded Paddle because they thought it might be a helpful tool to store their information, reflect on progress, and stay well following treatment. Nearly all respondents had used Paddle to check their latest scores on the questionnaires (95%), with the majority also checking how their scores had changed over time (89%), and reviewing their progress using the monthly prompt questions (61%). Three respondents reported minor technical problems experienced when logging into the app, but noted these were eventually resolved. No other technical problems were reported. When asked about the duration of the follow-up questionnaire period, 19% of respondents felt that 6 months was appropriate, 47% said they would prefer 12 months, and 34% felt the monthly questionnaire prompts should continue indefinitely.

Users’ agreement ratings with statements about Paddle are shown in Table 2. These reflected positive feedback regarding the feasibility, acceptability, and helpfulness of the app. Free-text comments highlighted the app’s role in supporting users to monitor their well-being, for example: “It helped me to keep an eye on myself following the end of therapy by completing the same questions I’d done beforehand,” “It’s helped me to check in regularly when life’s been busy and make sure the new habits I’d built in therapy had stuck,” “It’s been a great resource to keep my mental health a top priority,” “The app has been really helpful – and I am of the older generation who didn’t grow up with the internet and this technology, so that’s really a tribute to the Paddle app.”

**Table 2:** Number of Paddle users agreeing with statements about the app.

Statement	Strongly disagree	Disagree	Neutral	Agree	Strongly agree
Paddle has been straightforward to use	0	1	16	25	20
Paddle helped me to stay well	0	1	19	26	16
I would recommend Paddle to other people who’ve had treatment for anxiety or depression	0	1	16	27	18

## General Discussion

### Principal Findings

Taken together, the 2 studies reported here demonstrated preliminary evidence that the Paddle app is feasible to implement and acceptable to patients, with good potential to support patients during and after treatment and collect postdischarge outcome data. Feasibility was indicated by the successful use of Paddle within NHS TT services, with minimal technical problems evident across the stages of inviting participants, downloading and using the app, submitting data and integrating this with services. The app was also feasible from a risk management perspective, with services only needing to respond to indications of risk on 7 occasions.

Acceptability was indicated in Study 1 by the high uptake rate (66%) and use of the app as evidenced by patient and therapist feedback. Study 2 focused specifically on the postdischarge period, and thus invited participants at the point of discharge. Although appropriate for the study aims, this design meant participants had not been using the Paddle app to develop a set of useful therapy resources during treatment. They were therefore not familiar with the benefits of Paddle before being asked to use it to submit outcome data post discharge. Perhaps as a result, uptake was low (8%). However, despite the nonoptimal conditions for starting to use Paddle, 49% (156/321) of users submitted outcome data on the app and feedback suggested they found it acceptable to help monitor their well-being after treatment.

These studies provide an initial indication of the ways in which the app may have a positive impact on patients, though further studies are needed to examine this in more depth. Feedback highlighted the potential role of the app in supporting patients to organize and remember their thoughts from each session, keep their therapy materials together, and help patients to self-monitor their well-being after treatment had ended. Using the observed uptake rate of 66% (111/168) at the start of treatment, and the follow-up data collection rate of 49% (156/321), a tentative estimate would be that if used in routine practice, the app would be collecting follow-up data on around 32% of patients in NHS TT services. This is considerably higher than typically collected in the participating services (10%) and in these services nationally (1.3%). Although the findings demonstrate that not all patients will choose to use an app or to complete questionnaires after discharge, this suggests Paddle may offer a helpful route to improve follow-up data collection and understand more about how patients do after treatment.

Analyses indicated that the cohort of app users was representative of patients using the participating services regarding gender and age, though users from White ethnic backgrounds were overrepresented. The group of app users had an average of 13 treatment sessions and a good, reliable recovery rate (89/146, 61%), indicating the sample may have overrepresented those who did well in treatment. Although this provided a greater opportunity to demonstrate reliable relapse in the present study, future research could explore ways to broaden the use of Paddle to reach those who received fewer sessions and those from a wider range of ethnicities.

Although the present follow-up period of 6 months in Study 2 was slightly shorter than in other studies, the observed 36% relapse rate among those who reliably recovered during treatment is consistent with previous literature, falling between existing estimates of 29% [11] and 53% [12]. When analyzing all Paddle users together, regardless of their outcomes during treatment, 40% (63/156) showed reliable deterioration at any point during the follow-up phase. The data suggested that some of these increases in symptoms were not sustained, resolving later during follow-up, but this was only possible to explore where a subsequent timepoint was available. It does suggest, however, that some observed relapses or symptom deteriorations are short-lived, and may reflect natural fluctuations that are qualitatively different from more sustained relapses where additional support may be more beneficial.

We also found considerable evidence of posttreatment stability in Study 2, with a total of 74% (66/89) of those who reliably recovered during treatment remaining reliably recovered or reliably improved across the 6-month follow-up period. Similarly, among the cohort of patients who had reliably improved during treatment, 73% (86/118) remained so across the follow-up period. These findings are in line with the broader CBT literature, suggesting clinical gains are well-sustained following treatment [7-9]. It is also notable that 5 cases who reliably improved during treatment went on to reliably recover during follow-up, and in total, 20% (31/156) of all cases showed reliable improvement at one or more follow-up timepoints compared to their end-of-treatment scores. This demonstrates that further clinical gains can occur after treatment ends.

Employment data indicated that most Paddle users were in employment at the start of treatment and remained so throughout follow-up. Only 11 were unemployed at the start of treatment, but the majority of these moved into employment by the end of treatment and remained so across follow-up. These findings are therefore promising given the association between employment and well-being [33], though the extent to which they directly relate to NHS TT support is unclear and larger studies are needed.

### Limitations

These initial studies have some limitations to consider when interpreting the findings. First, although we made good efforts to elicit feedback from those who chose not to use Paddle, the extent of data here was limited, despite providing informative findings. Second, the uptake of the app in Study 2 was low when considered as a proportion of the total number of people invited. However, low uptake rates can be common among health apps with many contributing factors [34], and as discussed earlier, it is possible that, having completed treatment, patients were reluctant to start using the app to collect all their therapy materials together. A helpful next study would recruit participants at the start of treatment as per Study 1, allow users to build their therapy resources through treatment, then explore the rates of continued use and questionnaire completion post discharge. Third, although the sample of app users included people from a range of ethnic backgrounds, those from White backgrounds were overrepresented. Further work to support greater diversity among app users is needed. Fourth, the classification of clinical outcomes was only possible based on the available data. Although data availability was good, with 53% of users providing at least 3 datapoints, it is possible that certain evidence of stability or fluctuations in scores could not be demonstrated. Finally, as it was not possible to include a control group in Study 2, we cannot quantify the extent to which patients’ scores in the follow-up period were influenced by using Paddle to self-monitor their well-being, though participant feedback did suggest that Paddle was helpful in this regard. More broadly, it would be beneficial for studies in this area to examine postdischarge employment outcomes in greater detail alongside clinical outcomes. It would also be valuable to use methods that more clearly distinguish temporary lapses from more sustained episodes of relapse. The metric of reliable relapse developed for this study may offer a useful starting point for this.

### Conclusions

Overall, the results suggest that Paddle could offer a promising route toward improving support for patients both during and after treatment. Refinements such as further streamlining the risk management procedure are now possible due to the app having medical device status, and based on patient feedback, extending the follow-up period to 12 months may be appropriate. Future studies could helpfully explore users’ experience of the app in greater detail and examine factors associated with reliable deterioration or reliable relapse that may guide the provision of further support for these patients.

## Abbreviations

ADSM: anxiety disorder specific measures
CBT: cognitive behavioral therapy
DSM-5: Diagnostic and Statistical Manual of Mental Disorders, Fifth Edition
GAD-7: Generalized Anxiety Disorder 7-item version
iCT-PTSD: internet-delivered cognitive therapy for posttraumatic stress disorder
iCT-SAD: internet-delivered cognitive therapy for social anxiety disorder
ISO: International Organization for Standardization
NHS TT: National Health Service Talking Therapies
NICE: National Institute for Health and Care Excellence
PHQ-9: Patient Health Questionnaire 9-item version
PPI: patient and public involvement
WSAS: Work and Social Adjustment Scale

## Data Availability

The datasets generated or analyzed during this study are not publicly available due to privacy and ethical restrictions but are available from the corresponding author on reasonable request.
